# Supplementary material for: The Impact of Growing Area on the Expression of Fruit Traits Related to Sensory Perception in Two Tomato Cultivars
Source: Int J Mol Sci. 2024 Aug 20;25(16):9015. doi: 10.3390/ijms25169015 (PMC11354283; doi:10.3390/ijms25169015)
Supplement: Supplementary file 1 [file ijms-25-09015-s001.zip › Supplemental figure S1.pdf]

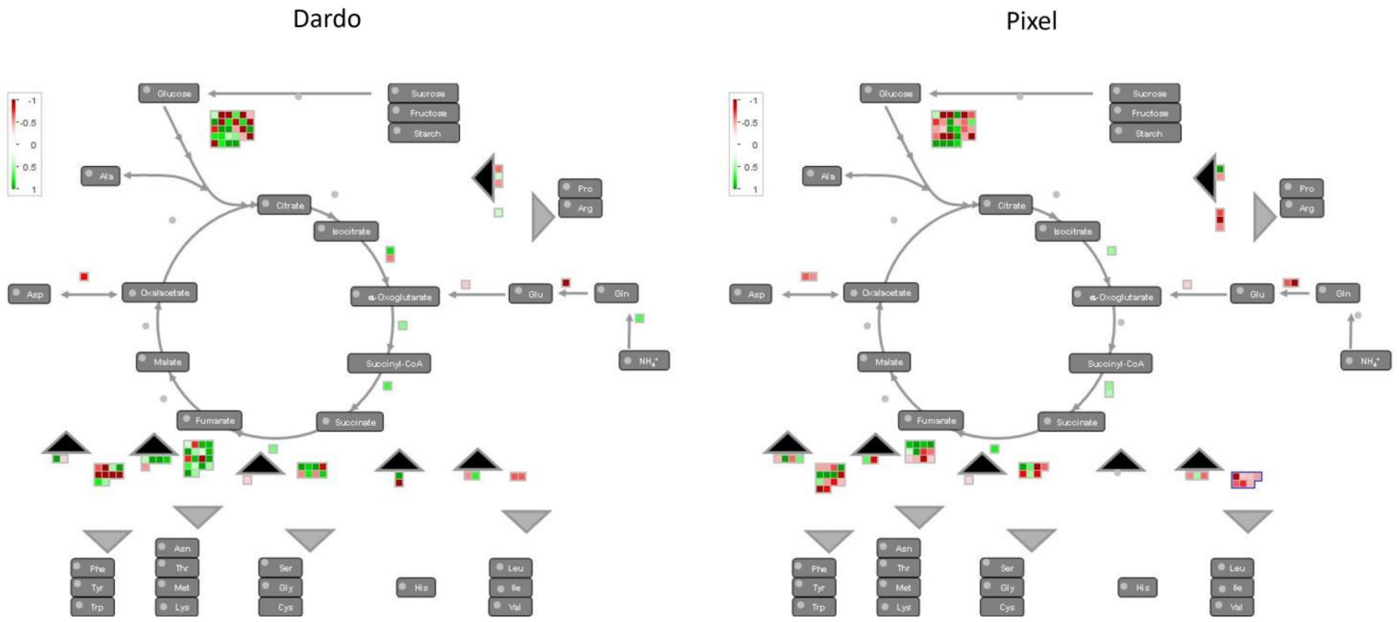

Figure S1: Differentially expressed gens (DEGs) involved in amino acid metabolism in Dardo (left panel) and in Pixel (right panel).
