# Supplementary figures and images for: The Impact of Growing Area on the Expression of Fruit Traits Related to Sensory Perception in Two Tomato Cultivars
Source: Int J Mol Sci. 2024 Aug 20;25(16):9015. doi: 10.3390/ijms25169015 (PMC11354283; doi:10.3390/ijms25169015)

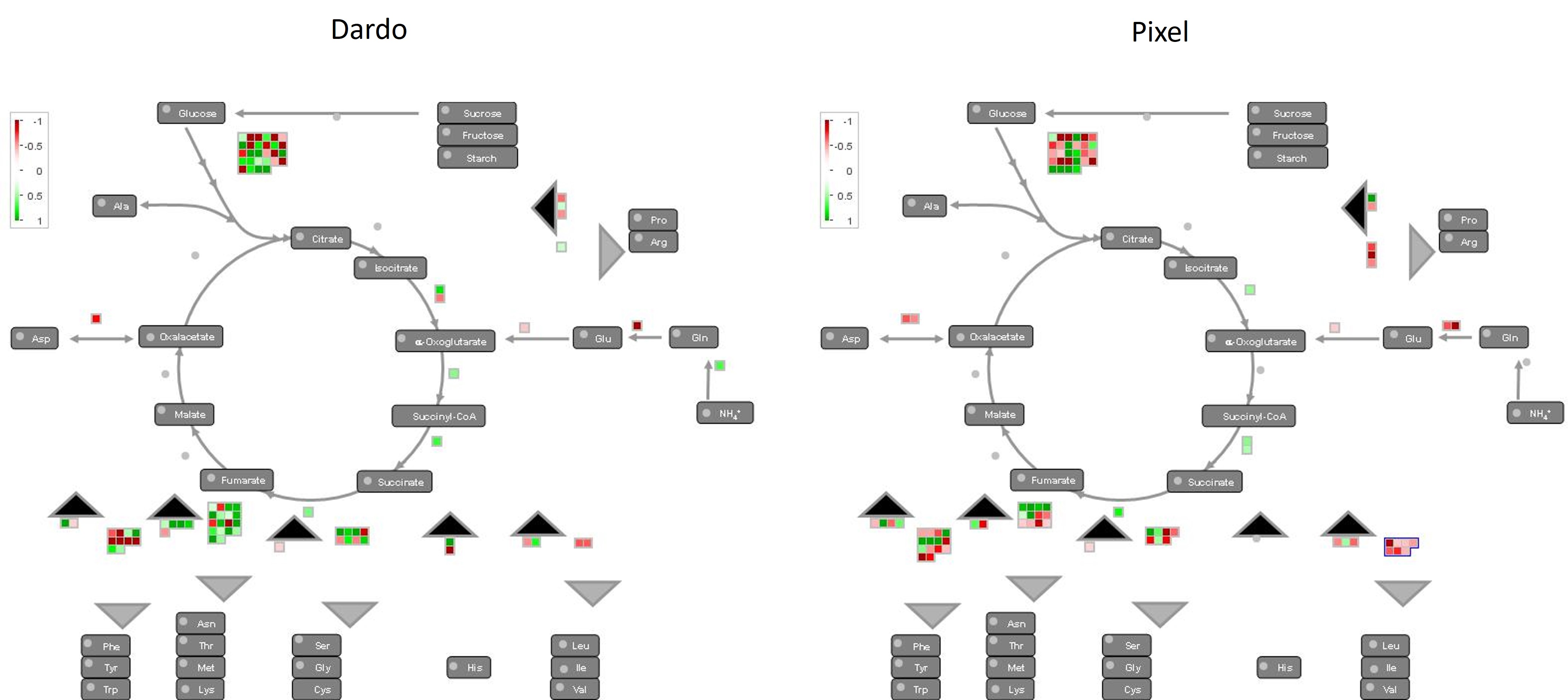

Supplement: Supplementary file 1 [file ijms-25-09015-s001.zip › Supplemental figure S1.jpg]
